# Prevalence of Comorbid Personality Disorder in Psychotic and Non-psychotic Disorders

**DOI:** 10.3389/fpsyt.2021.800047

**Published:** 2021-12-24

**Authors:** Qiang Wang, Lei Zhang, Jiechun Zhang, Zhihao Ye, Ping Li, Feng Wang, Yili Cao, Shaojun Zhang, Fang Zhou, Zisheng Ai, Nan Zhao

**Affiliations:** ^1^Department of Medical Statistics, Tongji University School of Medicine, Shanghai, China; ^2^Shanghai Pudong New Area Mental Health Center, Tongji University School of Medicine, Shanghai, China

**Keywords:** personality disorder, psychiatric disorders, comorbidity, prevalence, non-psychotic disorders

## Abstract

**Introduction:** The burden of personality disorders (PDs) in China is large and the focus on mental health services is increasing. However, there is a lack of sufficient evidence regarding the prevalence of comorbid PD in psychotic and non-psychotic disorders, and whether PDs have different distributions. We aimed to investigate the PD comorbidity distribution pattern between psychotic and non-psychotic disorders using a clinical population-based study.

**Materials and Methods:** We conducted a cross-sectional study of 1,497 patients in Shanghai. PDs were screened using the Personality Diagnostic Questionnaire Fourth Edition Plus (PDQ-4+). All patients were interviewed using the Structured Clinical Interview for Diagnostic and Statistical Manual of Mental Disorders, fourth edition (DSM-IV) Axis II (SCID-II). We compared the differences in PD comorbidities classified as the 10 types of PDs in the DSM-IV, in 531 patients with psychosis and 966 patients with non-psychotic disorders.

**Results:** More than one-third (37%) of patients with psychotic disorders met the criteria of at least one PD. Approximately half (46%) of patients with non-psychotic disorders met the criteria of at least one PD. Patients with non-psychotic disorders were more likely to meet the criteria of borderline (χ^2^ = 20.154, *p* < 0.001) and obsessive-compulsive PD (χ^2^ = 21.164, *p* < 0.001) diagnoses compared to those with psychotic disorders. In contrast, patients with psychotic disorders were more likely to meet the criteria of paranoid (χ^2^ = 11.144, *p* = 0.001) and schizotypal PD (χ^2^ = 14.004, *p* < 0.001) diagnoses than those with non-psychotic disorders.

**Discussion:** PD comorbidity is common and comorbidity distribution pattern is varied in patients with psychotic and non-psychotic disorders, implicating the development of specific strategies that could screen and assess PDs in psychiatric clinical practice.

## Introduction

Personality disorder (PD) is a maladaptive personality trait that develops in childhood and usually endures throughout one's life. The prevalence of PD is ~3–10% in the global population (1). PDs frequently occur in patients with psychiatric disorders (2–4) such as schizophrenia (5, 6) and depression (7, 8). The relationship between PD and other psychiatric comorbidities is firmly established (9, 10). For example, schizotypal PD is strongly associated with psychosis and has been included as one of the subtypes of disorders with a high clinical risk of psychosis (11) (i.e., genetic risk and deterioration syndrome, GRDS) (12). Schizotypal PD has also been reported to be associated with bipolar disorder and several anxiety disorders (13). Moreover, borderline PD has been found to be strongly associated with mood disorders (14, 15), especially bipolar disorder (16). PD traits are associated with a higher prevalence of psychiatric disorders (4), and these traits are associated with different psychopathologies (17, 18).

The comorbidity with PDs increases the severity of psychiatric disorders, worsens the prognosis, and influences the response to pharmacotherapy and psychotherapy (19, 20). Comorbidity of psychiatric disorders and PD is associated with a greater functional impairment that affects treatment adherence; it is also associated with high costs to society and negatively influencing progress (21). PD comorbidity also makes it more difficult for clinicians to provide appropriate management options for psychiatric patients (22). For example, patients with schizoid or avoidant PD comorbidity are found to have fewer treatment gains and poorer treatment adherence. Patients with borderline PD comorbidity are found to have a greater risk of suicide attempts (23). Therefore, determining the prevalence of PDs in different psychiatric comorbidities may provide clinical insights into further potential treatments.

The comorbid presence of PDs with other psychiatric disorders constitutes a group of psychiatric patients with highly complex symptoms that are difficult to treat, resulting in significant risks and functional impairments in patients (24). The objective of this study was to determine the prevalence of PDs among psychotic and non-psychotic disorders in a relatively large clinical population. Given that patients with psychotic or non-psychotic disorders are at increased risk for comorbid PDs, we hypothesized that patients with non-psychotic disorders would be associated with an increased prevalence and incidence of PD in this population. Besides, we aimed to investigate the PD comorbidity distribution pattern between psychotic and non-psychotic disorders.

## Methods

### Study Design

We examined the prevalence of PDs through both self-reporting and structure interview methods in patients from the Shanghai Pudong New Area Mental Health Center. The patients were drawn from a consecutive clinical sample of adult patients created to evaluate the prevalence of PDs between January 10, 2014 and May 30, 2021. This article presents a detailed analysis of the extensive baseline measurements of PD assessments. Further details of this study have been described in detail elsewhere (25). In brief, this was a single-site study and included outpatients and inpatients that (1) were aged 18–60 years; (2) had completed at least six years of education and were able to understand the study; (3) were willing to understand their own personality problems; and (4) had stable treatment conditions. The study was conducted in compliance with the Helsinki Declaration and was approved by the ethical committees of the Shanghai Pudong New Area Mental Health Center, Tongji University School of Medicine. To obtain consent, detailed oral and written information was provided to the patients and their informants to ensure that the patient fully understood the potential risks and benefits of the study. We confirm that we have read the Journal's position on issues involved in ethical publication and affirm that this work is consistent with those guidelines.

### Sample

For the current analysis, we selected a sample (*N* = 1,497) with psychotic and non-psychotic disorders (see Table 1). Using their medical records, it was determined that there were 531 patients with psychotic disorders who were diagnosed with schizophrenia, acute and transient psychosis, or delusional disorders. There were 966 patients with non-psychotic disorder who were diagnosed with depression, generalized anxiety disorder, panic disorder, social anxiety disorder, and obsessive-compulsive disorder. Patients with a history of stroke, brain trauma, central nervous system infection, severe cognitive impairment, or seizures were not included. The major diagnoses were schizophrenia, mood disorders, and anxiety disorders. A total of 236 (15.8%) patients were excluded because their diagnoses were unclear, such as general paranoid states, depressive states, or stress states.

**Table 1:** Demographic and clinical characteristics in patients with psychotic and non-psychotic disorders.

**Variables**	**Overall**	**Psychotic**	**Non-psychotic**	**Comparison *t/χ^2^ p*-value**	
Cases [n(%)]	1,497	531	966	–	–
Age(years) [Mean (SD)]	30.5 (9.5)	29.7 (9.1)	31.0 (9.6)	2.555	**0.011**
Age range (years)	18–60	18–60	18–60	–	–
Female [n(%)]	840 (56.1)	303 (57.1)	537 (55.6)	0.302	0.583
**Marriage [n(%)]**					
Single	818 (54.6)	363 (68.4)	455 (47.1)	64.702	**<0.001**
Married	575 (38.4)	138 (26.0)	437 (45.2)		
Divorced	67 (4.5)	22 (4.1)	45 (4.7)		
Widowed	37 (2.5)	8 (1.5)	29 (3.0)		
If unemployed [n(%)]	239 (16.0)	128 (24.1)	111 (11.5)	40.642	**<0.001**
If religious belief [n(%)]	412 (27.5)	159 (29.9)	233 (26.2)	6.012	**0.014**
**Education**					
Middle school [n(%)]	266 (17.8)	89 (16.8)	177 (18.3)	35.956	**<0.001**
High school [n(%)]	528 (35.3)	239 (45.0)	289 (29.9)		
College or higher [n(%)]	703 (47.0)	203 (38.2)	500 (51.8)		
**Personality character [n(%)]**					
Introversion	643 (43.0)	295 (55.6)	348 (36.0)	53.954	**<0.001**
In-between	597 (39.9)	170 (32.0)	427 (44.2)		
Extroversion	257 (17.2)	66 (12.4)	191 (19.8)		
Family History [n(%)]	157 (10.5)	59 (11.1)	98 (10.1)	0.341	0.559
**Diagnosis [n (%)]**					
Schizophrenia	470 (88.5)	470 (88.5)	–	–	–
Depression	419 (43.4)	–	419 (43.4)		
Anxiety disorder	301 (31.2)	–	301 (31.2)		
Obsessive-compulsive disorder	59 (6.1)	–	59 (6.1)		

*Bold in significant*.

### Measures

PDs were assessed using a self-report questionnaire and a clinical interview. The patients were selected using a two-stage probability sample design. In the first stage, all eligible patients were screened using a self-report questionnaire, which was conducted by nurses. In the second stage, the patients subsequently attended a clinical structure interview by trained psychiatrists.

The Personality Diagnostic Questionnaire-4+ (PDQ-4+) (26) has been widely used as a screening instrument for the identification of PDs in clinical populations (27, 28). The PDQ-4+ is a self-report, assessing 10 specific PDs in the fourth version of the Diagnostic and Statistical Manual of Mental Disorders (DSM-IV). The Chinese version of the PDQ-4+ has been validated to have a high sensitivity (0.89) and moderate specificity (0.65) for screening PD patients (4, 18). The PDQ-4+ consists of 107 true-false items, including four “too good” questions to prevent participants from undermining the problems and two “suspect questions” to determine whether participants are lying or responding insincerely. Except for the four “too good” and the two “suspect questions,” “yes” responses to the remaining 93 questions were regarded as pathological responses and counted as one point each. Higher subscale scores indicated a greater likelihood of having a certain type of personality disorder. The PDQ-4+ scores ten PDs: paranoid, schizoid, schizotypal, histrionic, narcissistic, borderline, antisocial, avoidant, dependent, and obsessive-compulsive. Each PD subscale is scored in several items with “yes” or “no” questions related to the DSM-IV PD diagnosis criteria. Higher PD scores indicate a higher severity of specific traits (29). PD subscales with a composite score of ≥ 4 or 5 were considered clinically relevant.

The structured clinical interview for the DSM-IV Axis II (SCID-II) (22, 30) was also designed to measure all 10 PDs in the DSM-IV, the criteria used for PD diagnoses in this study. The Chinese version of the SCID-II was translated and implemented (31). Previous studies have demonstrated that the SCID-II Chinese version has a relatively high test–retest reliability of 0.70, with a median coefficient for internal consistency of 0.70, which is highly consistent (0.90) with the clinical diagnoses.

All the clinicians in the psychiatric clinics were notified that there was an investigation on DSM-IV PDs in these patients, but they were only asked to diagnose mental disorders based on the routine clinical practice using the ICD-10. The clinical diagnoses of participants reported in this paper were mainly classified according to patients' medical records.

### Statistical Analysis

The results were first examined for missing data. To reduce missing data, the researchers checked the data of each patient when they completed the self-report questions and interviews to avoid missing data. The prevalence data of PD were presented as frequencies and percentages of the total sample of patients with psychotic or non-psychotic disorders. Before applying parametric statistics, we have checked the normality of the score's distribution. Univariate between psychotic and non-psychotic group differences were evaluated with unpaired-samples *t*-tests for normally distributed variables. Categorical variables were evaluated using the χ^2^ test. Descriptive variables were reported as means and standard deviations for normally distributed variables. Results were considered statistically significant at *P* < 0.05. Statistical analyses were performed on IBM SPSS version 21.0 (IBM Corp., Armonk, NY, USA).

## Results

The demographic and clinical characteristics of the patients with psychotic or non-psychotic disorders (*N* = 1,497) are shown in Table 1. No differences were found in terms of gender or family history between the two groups. Compared to the non-psychotic group, the patients in the psychotic group were younger, were more likely to be single, unemployed, and have religious beliefs, had lower educational levels, and had a greater proportion of introversion personality characteristics.

### The Prevalence of the PDQ-4+ Self-Reported PDs

**Figure 1:**
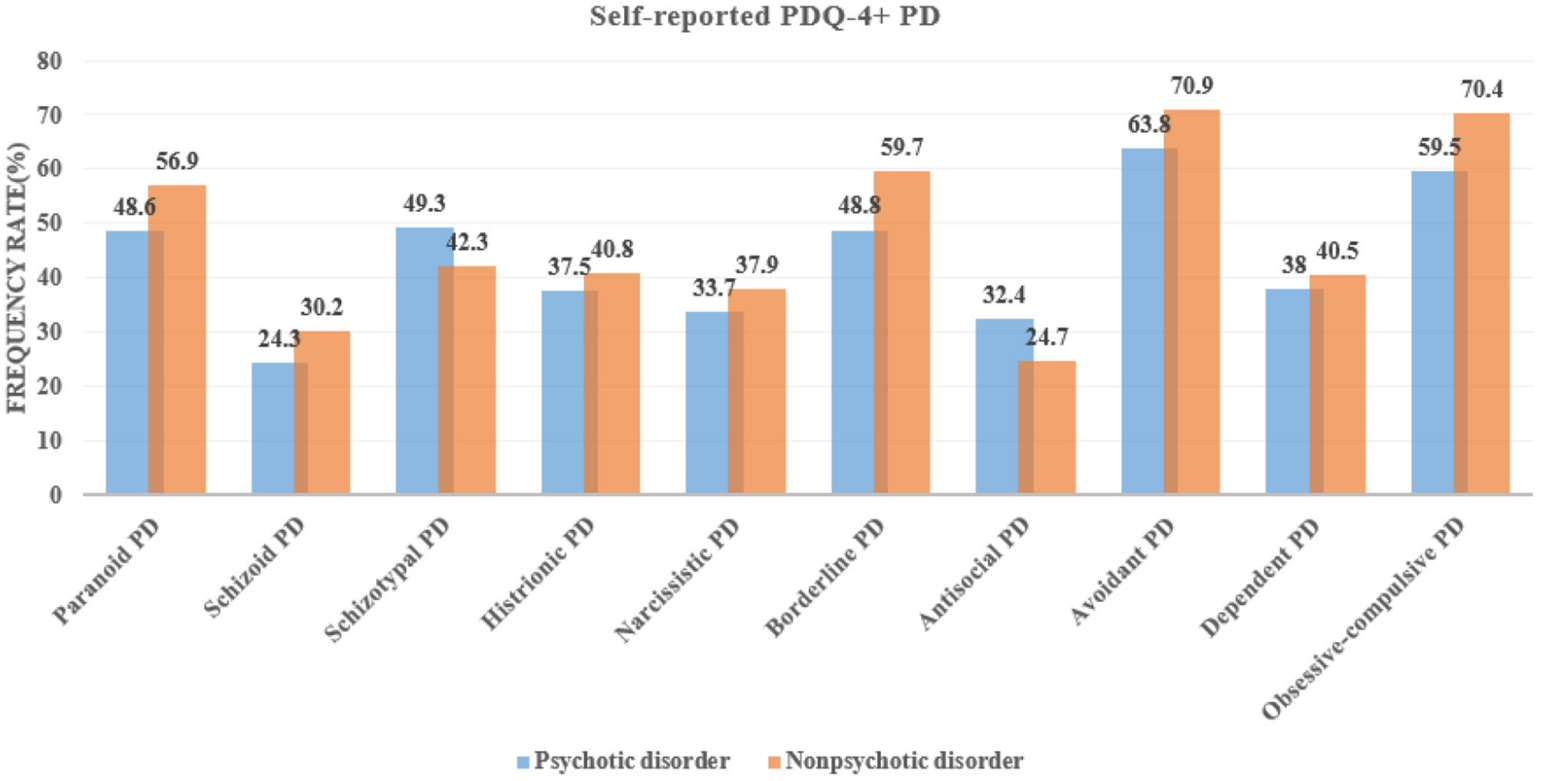
Prevalence of the fourth plus version of Personality Diagnostic Questionnaire (PDQ-4+) self-reported personality disorders (PDs) among patients with psychotic and non-psychotic disorders.

As shown in Figure 1, the first set of prevalence rates focused on self-reported PD traits from the PDQ-4+ questionnaire. Patients with psychotic disorders were commonly reported to have avoidant, obsessive-compulsive, schizotypal, borderline, and paranoid PDs (prevalence rate >40%). Similarly, except in different orders, patients with non-psychotic disorders were also commonly reported as having avoidant, obsessive-compulsive, borderline, paranoid, and schizotypal PDs. Patients with non-psychotic disorders reported more borderline (*t* = 3.503, *df* = 1495, *p* < 0.001), histrionic (*t* = 2.671, *df* = 1495, *p* = 0.008), narcissistic (*t* = 2.861, *df* = 1495, *p* = 0.004), avoidant (*t* = 3.194, *df* = 1495, *p* = 0.001), and obsessive-compulsive (*t* = 3.088, *df* = 1495, *p* = 0.002) PD traits than those with psychotic disorders, but not more schizotypal (*t* = 2.300, *df* = 1495, *p* = 0.022) and antisocial PD traits (*t* = 3.373, *df* = 1495, *p* = 0.001).

### The Prevalence of the SCID-II Diagnosed PDs

**Figure 2:**
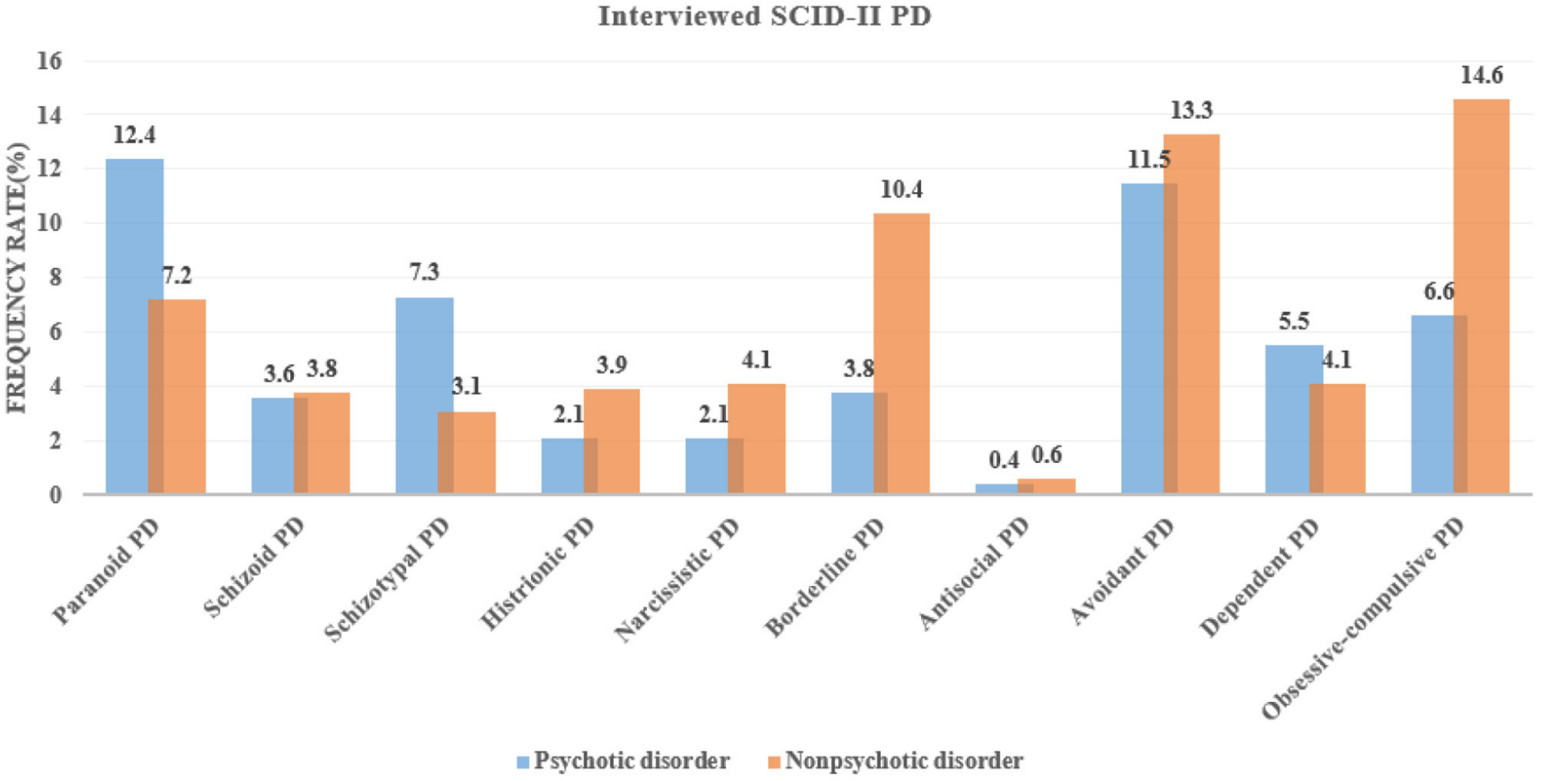
Prevalence of the structured clinical interview for DSM-IV Axis-II (SCID-II)-diagnosed personality disorders (PDs) among patients with psychotic and non-psychotic disorders.

When the structured interview tool of the SCID-II was used to determine PD diagnoses, the prevalence of paranoid and avoidant PDs was the highest in patients with psychotic disorders, while the prevalence of obsessive-compulsive, paranoid, and borderline PDs highest in patients with non-psychotic disorders (prevalence rate >10%). Patients with non-psychotic disorders were more likely to meet the criteria of borderline (χ^2^ = 20.154, *p* < 0.001) and obsessive-compulsive PD (χ^2^ = 21.164, *p* < 0.001) diagnoses than those with psychotic disorders. In contrast, patients with psychotic disorders were more likely to meet the criteria of paranoid (χ^2^ = 11.144, *p* = 0.001) and schizotypal PD (χ^2^ = 14.004, *p* < 0.001) diagnoses than those with non-psychotic disorders (Figure 2).

### Comorbidities

**Figure 3:**
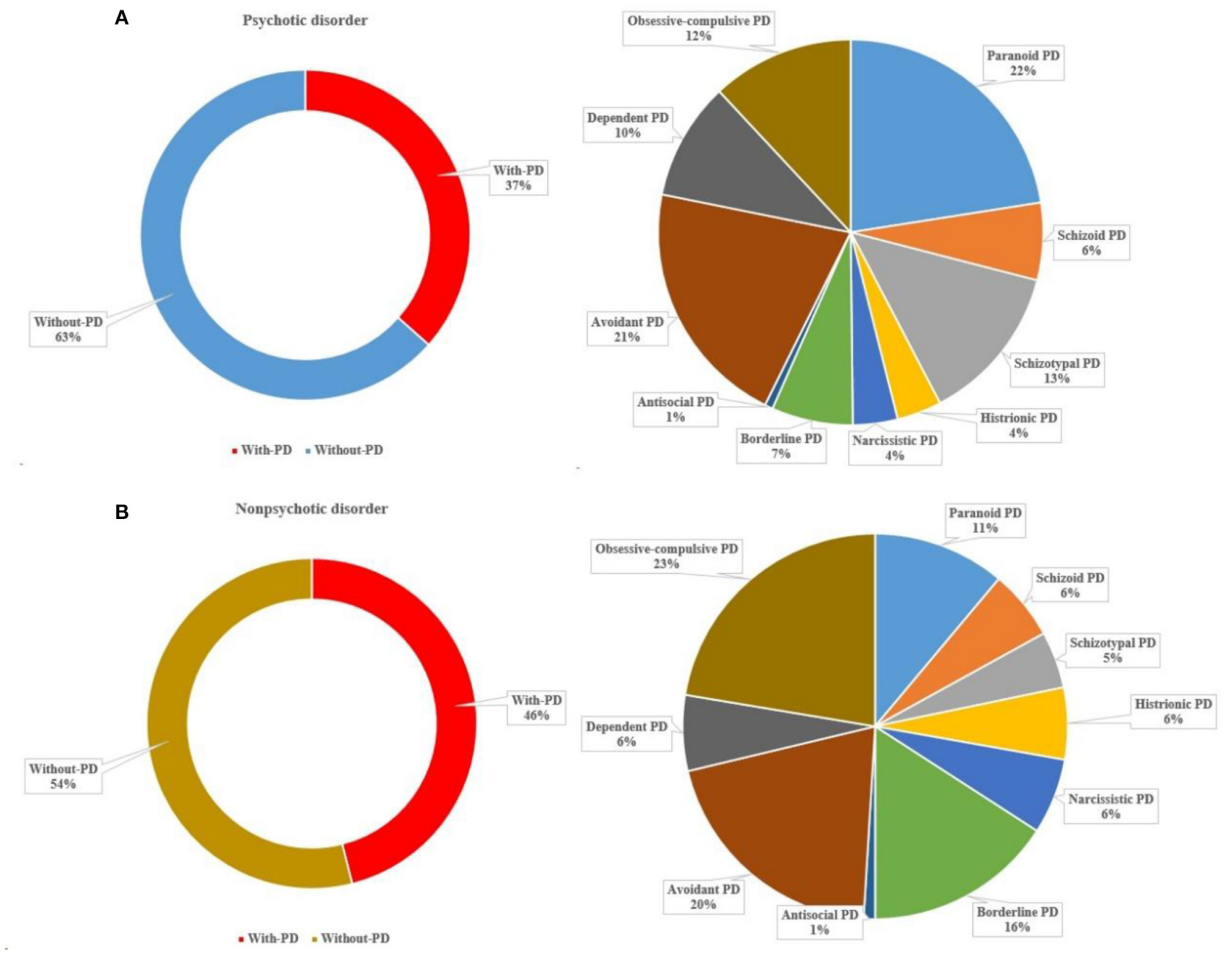
Comorbid PDs among patients with psychotic and non-psychotic disorders.

For patients with psychotic disorder, more than one-third (37%) met the criteria for at least one PD, with paranoid (22%), avoidant (21%), and schizotypal (13%) PDs accounting for the greatest proportion. For patients with non-psychotic disorders, approximately half (46%) met the criteria of at least one PD, with obsessive-compulsive (22%), avoidant (20%), and borderline (16%) PDs accounting for the greatest proportion (Figure 3).

## Discussion

This study recruited a large number of patients with psychotic and non-psychotic disorders in order to perform a comprehensive assessment of both self-reported PD traits and interview-determined PD diagnoses. We explored the prevalence and distribution differences of psychotic and non-psychotic comorbidities among the 10 specific types of PDs. For the self-reported PD traits, we found that both patients with psychotic disorders and non-psychotic disorders had the highest proportions of obsessive-compulsive and avoidant PDs. For the interview-determined PD diagnoses, patients with psychotic disorders had the largest percentage of paranoid and avoidant PDs, while patients with non-psychotic disorders had the highest proportion of obsessive-compulsive, paranoid, and borderline PDs. Considering the psychotic and non-psychotic subgroup differences in PD prevalence, we observed significant borderline and obsessive-compulsive PD predominance in patients with non-psychotic disorders and paranoid and schizotypal PD predominance in patients with psychotic disorders. More than one-third met the criteria of at least one PD among patients with psychotic disorders and about half meet the criteria of at least one PD among patients with non-psychotic disorders.

These results likely reflect the epidemiological distribution of PDs, which is commonly comorbid with both psychotic and non-psychotic disorders in clinical populations. Three other studies have specifically reported PD prevalence data for patients in the Chinese population, with similar prevalence rates to those seen in the current study. In the study with identical methods (PDQ-4+ and SCID-II) by Zhang et al. (4) nearly one-fourth of outpatients with schizophrenia met the criteria for at least one PD, with the most prevalent PD being paranoid PD (7.65%), followed by avoidant PD (7.53%). They found that 42.18% of outpatients with mood disorders met the criteria for at least one PD. A main factor explaining the discrepancy in the prevalence rates determined in the two studies may lie in the different samples used for estimating the prevalence of PD. While the present study assessed PD in a sample with both outpatients and inpatients, the study by Zhang et al. assessed PDs among only outpatients, since the severity of inpatients was more serious than that of outpatients; therefore, the comorbidity rate of PD in inpatients was higher in our study.

Another study applied the same methods (PDQ-4+ and SCID-II) for PD assessments by Wei et al. (32) reported that 204 (24.0%) of 850 outpatients with schizophrenia in the stable phase met the criteria for at least one PD. Zheng et al. (3) reported that 258 (42.36%) of 609 outpatients with major depressive disorder were recognized as having at least one criterion for the diagnosis of PD. Different study designs can explain the differences in the prevalence values reported. For example, if the study design is a double-blind method, and the evaluator is not clear about the clinical diagnosis of the patient, the results of personality evaluation may be different, resulting in different comorbidity rates. Interestingly, all these studies reported that the comorbidity rates were relatively consistent. Previous studies have compared the proportion of several psychiatric comorbidities across different types of PD, and certain findings were in line with those in our study. For example, borderline PDs mostly co-occur with mood disorders (33, 34), and avoidant, dependent, and obsessive-compulsive PDs frequently accompany depressive disorders, anxiety disorders, and obsessive-compulsive disorders (35–37).

Furthermore, we found that patients with psychotic disorders had a significantly higher proportion of paranoid and schizotypal PDs; non-psychotic disorders were more often comorbid with borderline and obsessive-compulsive PDs. This could be explained by the fact that they reflect the same diagnostic characteristics of the PD subgroup and other psychiatric disorders. For example, affective instability and emotional liability in borderline PD and mood disorders may share the same symptoms and lead to dysregulation (38). They may involve the same underlying brain structure pathologies and functions that have been detected in these disorders. For example, schizotypal PD and schizophrenia share a significant functional decline in visual–spatial working memory (39), which may result in vulnerability to cognitive impairments, a feature widely reported in patients with psychotic disorders (40, 41).

The main limitation of our study is its cross-sectional design, which makes it difficult to investigate the order of occurrence between PD and other psychiatric disorders. To evaluate the longitudinal relationship between PDs and other psychiatric disorders, as well as to minimize the influence of misdiagnosis, follow-up studies of PDs and other psychiatric disorders are needed. Second, this study was conducted with a sample of patients who met the clinical criteria for psychotic or non-psychotic disorders; therefore, our findings cannot be generalized to whole populations of problematic or non-treatment-seeking individuals. Third, among patients, antipsychotic or antidepressant medication histories and information on patient compliance with related treatments were not available. Future research should examine the potential interactive contributions of these measures when conducting PD assessments. Fourth, another relevant limitation of our study is the recruitment bias, in which some types of PD patients, such as those with schizoid and avoidant PD, were less likely to participate. To mitigate this, we took considerable care to allow for frequent breaks during the assessments and spread out the assessments across multiple visits for patients who were not active enough. We believe that these steps allowed for a high study quality despite the recruitment and assessment challenges present in this population.

## Conclusion

PD is a serious problem among patients with psychotic or non-psychotic disorders. Clinicians working with psychiatric patients should consider comorbid PDs; therefore, this group of patients should routinely be assessed for PD comorbidities. Longitudinal studies examining the factors associated with PD development among psychiatric patients will be helpful for disentangling the causal relations between disorders and treating them with integrated approaches accounting for both personality pathologies and psychotic or non-psychotic symptoms.

## Data Availability Statement

The original contributions presented in the study are included in the article/supplementary material, further inquiries can be directed to the corresponding authors.

## Author Contributions

QW and LZ wrote the manuscript and performed the statistical analysis. JZ and ZY offered great assistance for data analysis and wrote the study protocol. PL, FW, YC, SZ, and FZ were responsible for clinical assessment of patients and the data entry. ZA and NZ reviewed the manuscript and offered constructive suggestions. All authors contributed to the article and approved the submitted version.

## Funding

This study was supported from Excellent Young Medical Talents Training of Health Commission of Pudong New Area (PWRq2020-49); Pudong New Area of Science and Technology Development Fund (PKJ2019-Y25); Shanghai Municipal Health Industry Clinical Research Project (20204y0194); Shanghai Pudong New Area Mental Health Center, Tongji University School of Medicine.

## Conflict of Interest

The authors declare that the research was conducted in the absence of any commercial or financial relationships that could be construed as a potential conflict of interest.

## Publisher's Note

All claims expressed in this article are solely those of the authors and do not necessarily represent those of their affiliated organizations, or those of the publisher, the editors and the reviewers. Any product that may be evaluated in this article, or claim that may be made by its manufacturer, is not guaranteed or endorsed by the publisher.
